# Acute prefrontal hemodynamic responses to intermittent theta burst stimulation correlate with current depression and episode recurrence: A cross‐sectional study

**DOI:** 10.1111/pcn.70066

**Published:** 2026-04-14

**Authors:** Minxia Jin, Adam W.L. Xia, Wanda M.W. Chau, Nancy M.X.Y. Shi, Penny P. Qin, Bella B.B. Zhang, Rebecca L.D. Kan, Alvin H.P. Tang, Tim T.Z. Lin, Sharie X. Wang, Dicky W.S. Chung, Frank Padberg, Georg S. Kranz

**Affiliations:** ^1^ Department of Rehabilitation Sciences The Hong Kong Polytechnic University Hong Kong China; ^2^ Shanghai YangZhi Rehabilitation Hospital (Shanghai Sunshine Rehabilitation Center), School of Medicine Tongji University Shanghai China; ^3^ Department of Psychiatry, Virtus Medical Hong Kong China; ^4^ Department of Psychiatry and Psychotherapy LMU University Hospital, LMU Munich Munich Germany; ^5^ DZPG (German Center for Mental Health), partner site Munich‐Augsburg Munich & Augsburg Germany; ^6^ Mental Health Research Center The Hong Kong Polytechnic University Hong Kong China

**Keywords:** concurrent rTMS/fNIRS, major depressive disorder, neuroplastic responses, recurrence risk

## Abstract

**Background:**

Mounting evidence has indicated that multiple major depressive disorder (MDD) episodes are correlated with brain morphometric changes that confer an increased recurrence risk. Functional abnormalities underlying this recurrent vulnerability remain underexplored. Acute neuroplastic responses to repetitive transcranial magnetic stimulation hold promise to identify such functional alterations.

**Methods:**

This study involved 121 adults, including 39 individuals with current MDD, 41 individuals with MDD in remission, and 41 healthy controls. Cross‐sectional changes in hemodynamic responses and functional connectivity induced by a single session of intermittent theta burst stimulation over the left dorsolateral prefrontal cortex (dlPFC) were measured using simultaneous functional near‐infrared spectroscopy and analyzed with linear mixed models. Hierarchical regression examined the relationships between the number of prior depressive episodes and observed functional changes in patient cohorts.

**Results:**

Stimulation instantly elevated deoxyhemoglobin levels in the stimulated dlPFC across all participants, with greater increases in patient cohorts *versus* controls, demonstrating a persistent disease effect regardless of prior episodes or remitted state. Poststimulation increases in oxyhemoglobin concentration were current depression‐dependent and associated with multiple episodes in remitted patients. Prefrontal networks were characterized by a transient reduction in global node degree during stimulation in all groups.

**Conclusions:**

Our findings align with clinical and preclinical evidence linking disrupted neurovascular function in the prefrontal cortex to depression pathophysiology. Poststimulation responses in the stimulated dlPFC, reflecting the current state and multiple prior episodes, show potential for aiding in differential diagnosis and indicating depression vulnerability. Longitudinal studies are needed to determine whether such neurological dysfunctions represent disease progression or a vulnerability biomarker.

Major depressive disorder (MDD) is a prevalent mental illness that severely restricts psychosocial functioning and decreases quality of life,^1^ incurring billions of dollars annually.^2^, ^3^ Approximately 50% of people who experience an initial depressive episode will develop a second one, with the risk of recurrence escalating following each occurrence. This episodic course imposes significant individual disability and economic costs.^4^ Therefore, understanding the mechanism underlying the vulnerability to subsequent depressive episodes is essential for mitigating both personal and societal burdens.^5^

Large‐scale meta‐analyses and neuroimaging research have frequently identified abnormalities in mood‐related neural circuits in MDD, with prominent findings being reduced volume and altered connectivity in cortical and limbic brain regions, such as the prefrontal cortex (PFC) and hippocampus.^6^, ^7^, ^8^ However, these neural deviations often appear modest,^9^ possibly due to the heterogeneity of the clinical population. Specifically, milder cases may dilute observed neurological abnormalities, as hippocampal and medial PFC volume reductions have been consistently found to positively correlate with greater depression severity,^10^ longer illness duration,^11^, ^12^ and a higher cumulative number of major depressive episodes (MDE).^11^, ^13^, ^14^ This suggests that neuropathological changes may progress with episodic recurrence but could also reflect a pre‐existing vulnerability that accelerates disease progression and enhances the risk of further depressive episodes.^13^, ^15^ Therefore, investigating persistent and progressive neuropathologies may offer valuable insights into the mechanisms underlying recurrence risk, informing future longitudinal research. However, the role of functional changes within these presumably pathological circuits in mediating recurrence vulnerability remains largely unexplored.

Neuroplasticity, a fundamental function of the brain, describes the ability of the nervous system to respond to stimuli adaptively from the micro to the macro level. Accordingly, changes in brain activity and connectivity induced by repetitive transcranial magnetic stimulation (rTMS) can be quantified through neuroimaging and neurophysiological techniques (e.g., functional magnetic resonance imaging, fMRI; functional near‐infrared spectroscopy, fNIRS; electroencephalography, EEG), and serve as indicators of macro‐scale neuroplasticity.^16^ Such neuroplastic indices might prove useful as prognostic or diagnostic predictors in depression.

The dorsolateral PFC (dlPFC) is a brain region crucial for working memory, planning, abstract reasoning, and the regulation of attention and emotion. Studies have shown that depressed patients often exhibit atrophy of pyramidal neurons,^17^ decreased numbers of synapses,^18^ low GABA and glutamate levels^19^ and hypofunction in the dlPFC.^20^ Consequently, this brain area has been repeatedly targeted for rTMS assessment and treatment.^21^, ^22^ The acute neuroplastic responses provoked by rTMS applied to the dlPFC were confirmed to be correlated with daily mood^23^ and personality traits in healthy individuals,^24^ and to be predictive of treatment response in depression.^25^

In the present study, we combined a single‐session (600 pulses) of intermittent theta burst stimulation (iTBS), a variant of rTMS, with simultaneous functional near‐infrared spectroscopy (fNIRS) to measure short‐term, macro‐scale neuroplastic responses to stimulation. fNIRS measures brain activity and connectivity by detecting changes in blood hemoglobin concentration through neurovascular coupling, and its signal exhibits a good spatial and temporal correlation with the blood‐oxygen‐level‐dependent (BOLD) responses from fMRI.^26^ Most importantly, as an optical modality, fNIRS is less susceptible to rTMS‐generated electromagnetic interference, making it particularly suitable for integration with rTMS protocols.

In this study, we applied the concurrent rTMS/fNIRS paradigm to investigate the acute response to iTBS in terms of changes in hemoglobin concentration and connectivity across adults with current MDE (cMDD), adults with MDE in remission (rMDD) and healthy individuals (Controls), as well as to assess how such changes are associated with the most prominent recurrence risk predictor (i.e., the number of MDEs).^27^ Considering the deficits in cortical inhibition and excitation^28^ and disrupted neurovascular coupling in MDD,^29^ we hypothesized that (i) currently depressed individuals would demonstrate impaired hemodynamic response patterns and/or altered connectivity relative to controls, while individuals with rMDD would exhibit a similar trend of change, but to a lesser extent; and (ii) the number of previous episodes would be correlated with brain functional changes in depressed and remitted individuals.

## Methods

### Participants

Adults with current or prior MDD were recruited through the Hong Kong Integrated Community Centre for Mental Wellness and by generating publicity in a range of social media platforms. Healthy controls were recruited through advertisements with gender and age (±3 years) matched to the clinical groups. Participants were initially screened through phone interviews, with medical records reviewed to validate diagnoses and medication history. Participants deemed eligible were scheduled for a face‐to‐face clinical assessment (see Table S1 in the Supplement for details on inclusion and exclusion criteria). This study was registered at clinicaltrials.gov (NCT06402422) and conducted in accordance with the Declaration of Helsinki, with ethical approval from the Institutional Review Board of the Hong Kong Polytechnic University (HSEARS20230705001). Data collection took place from October 8, 2023, to December 18, 2024. All participants provided written informed consent and were financially compensated at a rate of 100 HKD (~12.8 USD) per hour for their time in the lab. The study was reported according to the STROBE statement.

### Procedures

Patient diagnoses were ascertained by a psychiatrist and research personnel during the clinical assessment, using the Chinese version of the Mini‐International Neuropsychiatric Interview.^30^ The severity of symptoms was assessed using the Hamilton Depression Rating Scale‐17 and the Patient Health Questionnaire‐9. To quantify the medication load, we adopted the method proposed by Zaremba et al. (2018).^31^ The overall medication load score was calculated for each participant by summing the individual medications. Cognitive ability was assessed by Raven's advanced progressive matrices,^32^ a 36‐item measure of nonverbal fluid intelligence. Participants had 40 min to answer as many items as possible in a quiet space free from distraction, with scores reflecting the number of correct responses.

The concurrent iTBS/fNIRS measurement followed established procedures.^24^ iTBS was delivered using a figure‐of‐eight‐shaped cooling coil (Cool‐B65) connected to a MagProX100 magnetic stimulator (MagVenture, Denmark). Each participant underwent a single iTBS session, which contained 20 trains of ten three‐pulse bursts at 50 Hz, delivered at a frequency of 5 Hz for 2 s with an 8‐s intertrain rest period, totaling 600 pulses. Stimulation was applied over the left dlPFC at 90% of the resting motor threshold (rMT), a level supported by recent concurrent TMS/fMRI studies^33^, ^34^ demonstrating that sub‐threshold intensities, especially 80% and 90% rMT, are sufficient to elicit robust dlPFC activation while minimizing sensory discomfort and ensuring participant compliance. The rMT was determined in accordance with standard practice^35^ by producing motor evoked potentials ≥50 μV in five out of ten consecutive trials in the right first dorsal interosseous muscle at rest.

**Fig. 1:**
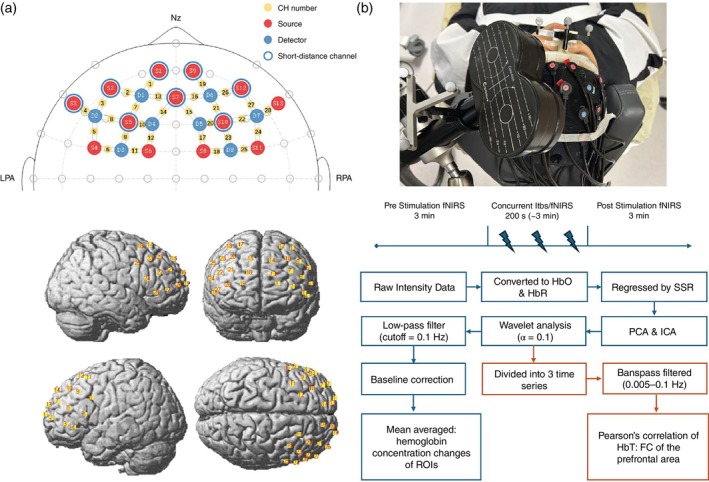
Schematic depiction of the measurement and analysis protocol for assessing hemodynamic responses and functional connectivity. (a) *Upper panel*: Cerebral hemodynamics were recorded from the prefrontal cortex using functional near‐infrared spectroscopy (fNIRS). The montage illustrates the placement of 13 light sources (red) and 8 detectors (blue), forming 28 measurement channels with a 3 cm distance shown as yellow edges; short‐separation channels are represented as blue circles. *Lower panel*: Diagram showing the standardized channel locations. (b) *Upper panel*: Concurrent transcranial magnetic stimulation/fNIRS setup. The fNIRS cap was arranged following the predefined montage. A 3D‐printed reference holder was securely positioned on the participant's nose to facilitate TMS navigation while the fNIRS cap was in place. Prior to testing, participants were seated comfortably in a reclining chair with their eyes closed. *Middle panel*: fNIRS data collection began once signal stability was confirmed, with data recorded over a timeline of 3 min before, approximately 3 min during, and 3 min after stimulation. *Lower panel*: Overview of the data preprocessing pipeline performed at the individual level.

Cortical hemodynamic signals were recorded before, during and after iTBS using a multichannel continuous‐wave NIRx Borealis system (NIRx Medical Technologies, Germany) at a frequency of 6.26 Hz. The optode montage consisted of 13 sources and eight detectors covering the bilateral prefrontal cortex according to the international 10–20 system (Fig. 1a). One short‐channel coupler provided eight short separation channels (SSC) was used to estimate scalp hemodynamics. Standard 3‐cm optode distances were maintained by plastic spacers for long channels, allowing for a penetration depth of 1.5 cm in the brain cortex.^36^

The TMS coil was positioned over the left dlPFC (Montreal Neurological Institute coordinates: −38, 44, 26; BA46)^37^ using a neuronavigation system (LOCALITE, Bonn, Germany) with fNIRS optode placement. Test stimulation at 90% rMT was applied to assess participant's tolerance to discomfort caused by TMS prior to fNIRS data acquisition. All fNIRS signals were recorded in a quiet room with dim lighting. Participants reclined comfortably in a chair with their eyes closed and were instructed to stay awake without any movement throughout the acquisition. Adverse effects were documented following the acquisition of fNIRS signals.

A Patriot 3D digitizer (Polhemus, Colchester, Vermont) was used to localize the anatomical locations of each optode in relation to standard head landmarks for each cap size. The MNI coordinates for each channel were obtained using the NIRS_SPM toolbox.^38^, ^39^ The anatomical locations of each channel are illustrated in Figure 1a. Channels having 80% or more probability of being located in the bilateral dlPFC (BA46) were included for further analysis. Accordingly, in the 56 cm cap, Channels 7 and 21 acted as anatomical markers for the left and right dlPFC, whereas Channels 2 and 26 were designated for the 54 cm and 58 cm caps.

### Preprocessing of fNIRS signals

The Homer2 toolbox and custom scripts written by the authors in MATLAB (version R2022a, The MathWorks, Natick, MA, USA) were used to preprocess the fNIRS data. First, the signal quality was checked by examining the cardiac power of each channel using spectrum analysis.^40^ Participants were excluded if the channel covering the dlPFC or more than 50% of channels exhibited bad quality. The preprocessing pipeline was previously established and implemented in our research.^24^ In brief, raw signals were first converted into optical density and then into relative concentration signals of oxyhemoglobin (ΔHbO) and deoxyhemoglobin (ΔHbR) based on the modified Beer–Lambert Law.^41^ The ΔHbR and ΔHbO datasets were processed independently. Short‐separation regression was performed by selecting the most correlated SSC sequence for each channel.^42^ Signal dimensionality was reduced by principal component analysis, retaining at least 99% of data variance.^43^ Independent component analysis was then applied to remove U‐shaped or inverted‐U‐shaped components^44^ and transient fluctuations coinciding with each burst of TMS pulses. Residual artifacts were removed using wavelet‐based denoising with a threshold of *α* = 0.1,^45^ and the corrected time‐series data were reconstructed for subsequent preprocessing. Physiological signals were filtered using a low‐pass filter with a high‐frequency cut‐off at 0.1 Hz. Low‐frequency signals were preserved to capture the cumulative effects of iTBS. Baseline correction was performed using the mean signal calculated from the 3‐min resting state prior to stimulation. Hemoglobin concentration changes during and after iTBS were averaged and included in the statistical analysis. See Appendix S1 in the Supplement for further preprocessing details.

### Characterizing functional networks of the prefrontal cortex

The wavelet‐denoised hemoglobin concentration signals were bandpass filtered (0.005–0.1 Hz, 2nd‐order Butterworth). The total hemoglobin (HbT) signal was derived from summing HbO and HbR. Correlations between such HbT time series across all channel pairs for each phase (pre‐, during‐, and poststimulation, ~200 s each) were quantified *via* Pearson correlation coefficients (*r*_*ij*_). Negative correlations and nonsignificant connections (*P* ≥ 0.05, uncorrected) were set to zero, generating weighted (*r*‐values, *w*_*ij*_) and binarized (0/1, *b*_*ij*_) adjacency matrices; this thresholding method presumes that only statistically significant positive correlations reflect genuine functional interactions.^46^

Local functional connectivity was evaluated using weighted Pearson coefficients (*w*_*ij*_), with significant correlations Fisher z‐transformed by the atanh function to normalize the distribution for group‐level comparison. Next, graph theory metrics were calculated to describe the topology of functional networks,^47^ with nodes as channels and edges as significant connections. Given the small size of nodes within the network, we limited the analysis to global metrics, including (i) normalized global connection strength ($w\bar{D}$); and (ii) normalized global node degree ($\bar{D}$), following methods outlined in^47^, ^48^ (See Appendix S2 for equations).

### Sample size and statistical analysis

The required sample size for this study was calculated using one‐way analysis of variance (G*Power software, version 3.1.9.7). Assuming a medium‐to‐large effect size (Cohen's f = 0.3), a significance level (*α*) of 0.05, and a statistical power of 0.8, the minimum total sample size required was determined to be 111 participants (37 per group). Considering an anticipated ~20% dropout rate due to intolerable pain during stimulation observed in our previous work,^49^ we planned to enroll 47 participants in each group.

Parameter statistical tests were used to capture group differences regarding continuous demographic and clinical characteristics only if the Q‐Q plot confirmed the normal distribution. Chi‐square tests compared categorical variables. The balance of age and gender distribution was evaluated using numerical diagnostics of standardized differences among the groups, with a cut‐off of 0.20 indicating acceptable balance.^50^

Linear mixed models (LMM) with an unstructured variance–covariance structure were employed to analyze hemodynamic changes (ΔHbO & ΔHbR) and global connectivity (wD̄ & D). The models included group, time, and their interaction as fixed effects, with a random intercept for participants. To account for potential confounding, pre‐stimulation mean Hb values were initially compared across groups using one‐way ANOVA; variables showing significant baseline differences were subsequently included as covariates in the LMM.

Regarding local connectivity, we conducted pairwise *t*‐tests between groups and time phases (pre‐, during‐, and poststimulation). The threshold for statistical significance was set at *P* < 0.05 following false discovery rate correction. The effect size was represented using Cohen's *f* ^2^, which measures the ratio of variance explained by the fixed effect relative to the unexplained variance. Cohen's *f* ^2^ ≥ 0.02, 0.15, 0.35 represent small, medium, and large effect sizes, respectively.^51^

Subsequently, correlation analyses were performed to investigate potentially confounding effects of medication load and symptom severity on significantly changed hemoglobin responses or connectivity indices. Hierarchical regression was then conducted within patient groups on indices that were not influenced by medication, using the number of episodes as a regressor of interest while controlling for common demographic variables (age, gender, and years of education).

## Results

### Study participants

A total of 1014 individuals registered for the study, with 143 participants enrolled (cMDD = 49; rMDD = 47; Controls = 47). The slight variation from the minimum calculated sample size of 47 per group resulted from a higher‐than‐expected dropout rate in the cMDD group. Eighteen participants withdrew because of intolerable pain perception during TMS test pulses, newly identified TMS contraindications, allergy to the fNIRS cap, or personal reasons. One dataset from the Controls was lost to a computer failure, and four participants were excluded due to excessive head movement during stimulation or bad signal quality, as described in the Methods (see Fig. S1 for recruitment flow); therefore, 39 cMDD, 41 rMDD, and 41 Controls were included in the final analysis. The participants' characteristics and distribution of psychotropic medication were summarized in Tables 1 and 2. Standardized mean differences for matched variables, age and gender distribution, ranged from 0.038 to 0.141 across groups, suggesting an overall acceptable balance in the final analyzed sample.

**Table 1:** Characteristics of the study participants

Characteristic	cMDD (*n* = 39)	rMDD (*n* = 41)	Controls (*n* = 41)	*P* value
Age, mean (SD)^†^	34.33 (12.98)	33.85 (12.04)	35.02 (12.54)	0.913
*N* of male (%)^†^	9 (23.01)	10 (24.39)	12 (29.27)	0.798
*N* of right‐handedness (%)	35 (89.74)	38 (92.68)	38 (92.69)	0.844
Head circumstance, mean (SD)	55.57 (1.97)	55.71 (1.88)	54.99 (1.56)	0.175
*N* of Han Chinese (%)	39 (100)	38 (92.68)	39 (95.12)	0.248
Years of education, mean (SD)	15.49 (3.43)	16.55 (3.04)	16.27 (2.91)	0.297
Raven's APM	22.05 (5.76)	23.92 (5.84)	23.28 (6.41)	0.386
Occupational status				0.063
*N* of unemployed (%)	9 (23.08)	3 (7.31)	1 (2.44)	
*N* of employed & students (%)	30 (76.92)	27 (65.85)	38 (92.68)	
*N* of retired (%)	0	1 (2.44)	2 (4.88)	
Marital status				0.01*
*N* of single (%)	28 (71.79)	26 (63.41)	16 (39.02)	
*N* of married (%)	10 (25.64)	14 (34.15)	25 (60.98)	
*N* of divorced or widowed (%)	1 (2.56)	1 (2.44)	0	
Onset age, mean (SD)	23.12 (11.93)	25.66 (11.23)	/	0.333
PHQ‐9, mean (SD)	12.85 (5.37)	3.51 (2.77)	2.15 (1.81)	< 0.001*
HAMD‐17, mean (SD)	16.05 (5.05)	4 (2.77)	/	< 0.001
*N* of Single Episode (%)	19 (48.72)	23 (56.10)	/	0.509
*N* with comorbid conditions (%)	27 (69.23)	24 (58.54)	/	0.320
*N* with familial risk for affective disorders	14 (35.90)	12 (29.27)	0	0.527
Medication load, mean (SD)	1.41 (1.19)	0.50 (0.68)	/	< 0.001*
Resting motor threshold, mean (SD), %	49.13 (8.18)	48.95 (9.43)	48.67 (9.85)	0.527
Pain during iTBS (VAS), mean (SD)	5.67 (2.37)	5.79 (2.18)	5.73 (2.05)	0.972

^†^ Gender distributions varied across groups due to differing withdrawal profiles.

APM, advanced progressive matrices; cMDD, major depressive disorder with current episode; HAMD‐17, hamilton depression rating scale‐17; *N*, number; PHQ‐9, health questionnaire‐9; rMDD, remitted major depressive disorder; SD, standard deviation; VAS, visual analogue scale.

**Table 2:** Distribution of psychotropic medication

Medication	cMDD (*n* = 39)	rMDD (*n* = 41)
Antidepressants, No. (%)		
SSRIs	22 (56.41)	11 (26.83)
SNRIs	6 (15.38)	5 (12.20)
Tricyclic antidepressants	1 (2.56)	0
Tetracyclic antidepressants	1 (2.56)	0
Miscellaneous antidepressants	2 (5.13)	0
Phenylpiperazine antidepressants	3 (7.69)	0
Anxiolytics, No. (%)	10 (25.64)	2 (4.88)
Atypical antipsychotics, No. (%)	6 (15.38)	2 (4.88)
Other, No. (%)	8 (20.51)	0
No current medication, No. (%)	11 (28.21)	23 (56.10)

Each psychotropic medication was categorized as absent = 0, low = 1 (equal or lower average dose), or high = 2 (greater than average dose), relative to the midpoint of the daily dose range recommended by Physician's Desk Reference (https://www.pdr.net/).

cMDD, major depressive disorder with current episode; rMDD, remitted major depressive disorder; SNRIs, selective serotonin noradrenaline reuptake inhibitors; SSRIs, selective serotonin reuptake inhibitors.

### iTBS‐induced hemodynamic responses in the dlPFC

**Fig. 2:**
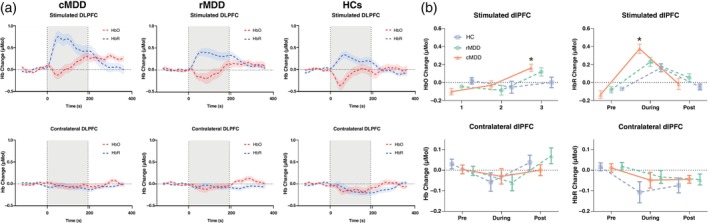
Hemodynamic responses results. (a) The time course of hemodynamic responses in the stimulated and contralateral dorsolateral prefrontal cortex (dlPFC) across participants with major depressive disorder (MDD), remitted MDD (rMDD), and healthy controls (Controls). Lines represent the mean, while the shaded areas denote ±1 standard error. (b) Mean HbO and HbR concentrations are presented across the pre‐, during‐, and poststimulation phases to visualize uncorrected values. Data are expressed as mean ± SEM. Asterisks denote significant differences derived from ΔHb analysis. (Bonferroni‐corr *P* < 0.05).

Traces of hemodynamic responses and *post hoc* are shown in Figure 2. The one‐way ANOVA revealed a significant difference in mean left dlPFC HbO across groups at baseline (*F*_2, 118_ = 3.63, *P* = 0.030). No other significant baseline differences were identified for the right dlPFC or HbR (Table S2).

Stimulated dlPFC HbO responses demonstrated a significant group‐by‐time interaction effect (*F*_4, 236_ = 2.647, *P* = 0.034, *f* ^2^ = 0.045), alongside significant main effects for group (*F*_2, 118_ = 4.157, *P* = 0.018, *f* ^2^ = 0.071) and time (*F*_2, 236_ = 5.630, *P* = 0.004, *f* ^2^ = 0.048). *Post hoc* tests showed that the cMDD group exhibited a significantly greater post‐iTBS ΔHbO compared with Controls (p_*corr*_ <0.001, 95% CI [0.127–0.521]), while the rMDD group showed a similar but nonsignificant trend (p_*corr*_ = 0.077). This interaction remained significant after controlling for baseline HbO (*F*_4, 236_ = 2.942, *P* = 0.021, *f* ^2^ = 0.050), with the cMDD group maintaining a significantly greater ΔHbO response (p_*corr*_ = 0.003, 95% CI [0.074–0.448]).

Similarly, stimulated dlPFC HbR showed a significant interaction effect (*F*_4, 236_ = 2.892, *P* = 0.023, *f* ^2^ = 0.050) and main effects for group (*F*_2, 118_ = 3.291, *P* = 0.041, *f* ^2^ = 0.066) and time (*F*_2, 236_ = 38.001, *P* < 0.001, *f* ^2^ = 0.323). This interaction was driven by the increased ΔHbR during stimulation in patient groups compared with Controls (cMDD, p_*corr*_ < 0.001, 95% CI [0.115–0.600]; rMDD, p_*corr*_ = 0.036, 95% CI [0.012–0.490]). Regarding the contralateral dlPFC, only significant time effects were observed for ΔHbO (*F*_2, 232_ = 3.975, *P* = 0.020, *f* ^2^ = 0.033) and ΔHbR (*F*_2, 232_ = 5.472, *P* = 0.005, *f* ^2^ = 0.047). *Post hoc* test only confirmed significant reductions in HbR both during (p_*corr*_ = 0.01) and after stimulation (p_*corr*_ = 0.019).

In the patient cohorts, neither medication load index nor symptom severity was correlated with hemodynamic responses that exhibited significant group effects (See Table S3 in Supplement).

### iTBS‐induced functional connectivity changes in the PFC

**Fig. 3:**
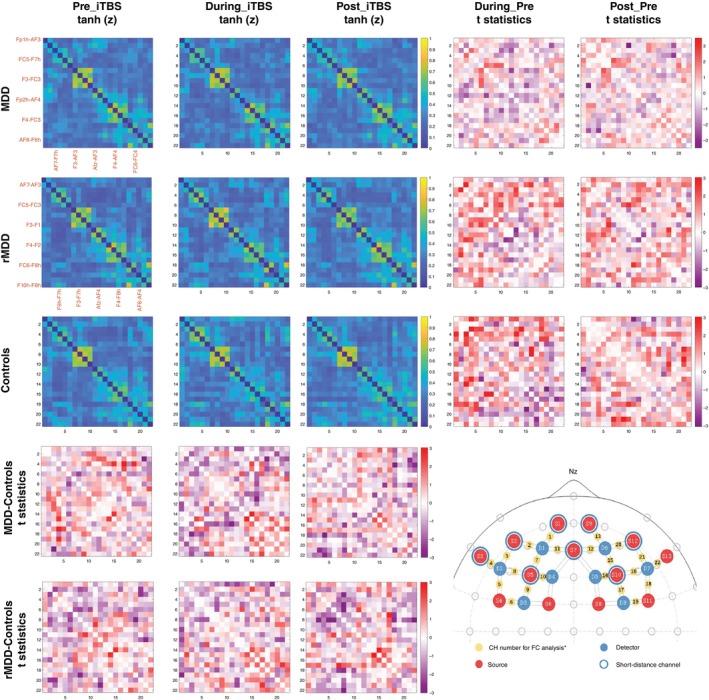
Statistical effects of Group and Stimulation Phase on local functional connectivity (FC). The figure illustrates the statistical effects of group differences (rows) and stimulation phase differences (columns) on local FC. Color‐coded grand averages of Fisher z‐transformed Pearson‐adjacency matrices are presented. The group contrasts (independent *t*‐statistics) and stimulation phase contrast (paired *t*‐statistics) are visualized as color‐coded matrices of *t*‐statistics derived from pairwise comparisons. Statistical t‐contrast matrices are scaled within the range of [−3, 3]. * Channels 11, 12, 14, 15, 17, and 18 were excluded from all datasets because of low signal quality in more than 50% of participants.

Channels 11, 12, 14, 15, 17, and 18 were omitted from all datasets, as more than half of the recordings showed poor signal quality in these channels. Local measures of functional connectivity are presented in Figure 3 as group averages. No significant change was observed (all *P* > 0.05, false discovery rate corrected); neither disease status nor stimulation influenced measures of local network connectivity.

In the case of global network metrics, global connection strength ($w\bar{D}$) was neither significantly different between groups nor time phases. Global node degree ($\bar{D}$) was only affected by stimulation (*F*_2, 236_ = 4.399, *P* = 0.013, *f* ^2^ = 0.037). The Bonferroni *post hoc* analysis revealed a significant reduction in $\bar{D}$ during iTBS (mean ± SE: 0.548 ± 0.011) relative to pre‐stimulation (0.574 ± 0.011, p_*corr*_ = 0.044).

### Relationships between dlPFC hemodynamic responses and number of prior MDEs

Given the absence of interaction or group effects in connectivity changes, the relationship between dlPFC hemodynamic responses and the number of prior MDEs was analyzed by hierarchical multiple regression (Table 3). To reduce recall bias in retrospective reports, we categorized the number of previous episodes following Treadway (2015) into three groups: single episode, 2 to 4 episodes, and five or more episodes.

**Table 3:** Hierarchical regression results predicting left dlPFC hemoglobin responses using number of episodes and demographic covariates in patient groups

	Demographic covariates (Beta)			Regressor of interest (Beta)	Statistics
	Age	Gender	Education years	Number of prior episodes	
HbR responses during stimulation (cMDD)					
Model 1	−0.010	−0.300	0.014		*R* ^2^ = 0.305, *F*_3,34_ = 1.163
Model 2	−0.009	−0.290	0.015	−0.095	*R* ^2^ = 0.314, *F*_4,33_ = 0.900, Δ*R* ^2^ = 0.005
HbR responses during stimulation (rMDD)					
Model 1	−0.017	0.549*	−0.014		*R* ^2^ = 0.500, *F*_3,37_ = 4.100*
Model 2	−0.017	0.548*	−0.014	−0.002	*R* ^2^ = 0.500, *F*_4,36_ = 2.992*, Δ*R* ^2^ = 0.000
HbO responses post stimulation (cMDD)					
Model 1	0.008	−0.130	−0.033		*R* ^2^ = 0.445, *F*_3,34_ = 2.801
Model 2	0.008	−0.121	−0.032	−0.083	*R* ^2^ = 0.461, *F*_4,33_ = 2.222, Δ*R* ^2^ = 0.014
HbO responses post stimulation (rMDD)					
Model 1	−0.007	−0.307*	−0.006		*R* ^2^ = 0.374, *F*_3,37_ = 2.009
Model 2	−0.004	−0.264*	−0.001	0.259*	*R* ^2^ = 0.566, *F*_4,36_ = 4.247*, Δ*R* ^2^ = 0.181

*Note:* *p < 0.05.

In the rMDD group, including the number of prior episodes as a predictor significantly explained poststimulation ΔHbO increases (*F*_4, 36_ = 4.247, *P* = 0.006), accounting for an additional 18.1% of the variance. The number of previous MDEs was a significant predictor (*β* = 0.259, *P* = 0.004), indicating that higher episode numbers were associated with greater HbO elevations. Sex differences were observed, with males exhibiting greater increases in HbR during stimulation and smaller increases in ΔHbO compared to females. In contrast, hemodynamic responses in the cMDD group were not significantly influenced by demographic factors or the number of prior episodes (all *P* > 0.05).

## Discussion

Using our developed concurrent rTMS/fNIRS paradigm, we investigated the short‐term neuroplastic effects of iTBS at the macroscale across adults with cMDD and rMDD in comparison with Controls. Key findings include: (i) iTBS over the left dlPFC elicited robust acute alterations in hemodynamic responses and functional connectivity, that is, increased ΔHbR in the stimulated region and decreased bilateral PFC connectivity during stimulation, as well as a sustained ΔHbR reduction in the contralateral dlPFC; (ii) Inter‐group differences emerged in the stimulated dlPFC, with patient participants showing a more pronounced ΔHbR increase than Controls, with cMDD also showing a significant poststimulation ΔHbO increase *versus* Controls; and (iii) In the rMDD group, but not cMDD, a history of multiple MDEs was associated with a greater post‐iTBS ΔHbO elevation.

Enhanced neural activity in the brain drives changes in local blood flow *via* neurovascular coupling,^52^ forming the physiological foundation for fNIRS. Typically, elevated ΔHbO and reduced ΔHbR are indicative of increased cortical excitation, which is widely thought to underlie the effects of high‐frequency rTMS through the LTP‐like mechanism.^53^ However, our results showed an inverse hemodynamic pattern in the stimulated dlPFC during concurrent iTBS/fNIRS, consistent with previous findings in an independent healthy cohort,^24^ and aligned with the assumed local brain activity changes driven by high‐frequency rTMS, as illustrated in a review.^54^ Supporting evidence comes from animal studies integrating 20 Hz rTMS with glucose‐based metabolic tracers, which reveal that the rTMS effects are pattern period‐dependent, with metabolic activity reduced during stimulation and increased after stimulation.^55^ Our inverse pattern may also involve vasoconstriction caused by stimulation. Pial arteries on the brain surfaces contain peri‐vascular nerves and multiple layers of smooth muscle cells with great contractile capabilities.^52^ Two‐minute electrical stimulation of the smooth muscle walls would rapidly reduce vessel diameter to its smallest size within approximately one minute,^56^ coinciding with the ΔHbR positive peak and the corresponding ΔHbO negative trough observed in our results. Additionally, finite element modeling and fNIRS measures of cerebrovascular reactivity to stimulation found that the pial arteries can be strongly affected by the high current density in the surrounding cerebrospinal fluid. Therefore, short duration stimulation (<three min) may mainly elicit a vascular response, potentially resulting in a prolonged initial dip (12.13 ± 3.8 s) in ΔHbO.^57^, ^58^

Observed inter‐group differences on local hemodynamic responses during and after stimulation might reflect the vascular‐ground MDD pathophysiology. Resting cerebral blood flow (CBF) reduction has been widely documented in the bilateral PFC^59^ with left‐hemispheric predominance.^60^, ^61^ Insufficient CBF may fall below the threshold required for normal brain oxygenation, leading to hypoxia^52^ and potentially accounting for the greater ΔHbR increase observed in the depressed individuals during iTBS in our study. The pronounced functional hyperemia following stimulation might thus serve as a compensatory response to this hypoxic state. Moreover, animal and postmortem human studies have revealed blood–brain barrier^62^ and endothelial dysfunction^63^ in the PFC associated with depression. Such vascular impairments disrupt the neurovascular coupling, compromise neuronal signaling, disturb regulation of brain temperature, and ultimately impair brain homeostasis.^29^ Although the precise mechanisms remain unclear, we assume that these alterations could render individuals with MDD more susceptible to exaggerated hemodynamic responses to external magnetic stimulation.

During stimulation, pronounced ΔHbR increases differentiated patients from healthy controls irrespective of current status, but were unrelated to either the number of prior MDEs or current symptom severity. This suggests a disease‐sensitive signal with limited evidence for progressive, episode‐accumulating effects. In contrast, poststimulation alterations in ΔHbO were more likely to represent pure neuroplastic responses. This signal appeared to be state‐dependent, as inter‐group differences were confined to currently depressed individuals and controls. rMDD participants displayed a similar but attenuated hemodynamic response to cMDD, consistent with observations from neuroimaging studies^9^, ^64^ that current depression states could heighten the neurobiological deviations from healthy norms. Notably, greater prior MDE within remission was linked to larger poststimulation ΔHbO increases, indicating a progressive disease effect or potential recurrence risk. Collectively, these heightened hemodynamic responses may reflect a long‐lasting neural homeostatic disruption, which, through complex interactions with environmental factors,^65^ contributes to emotional dysregulation and clinical symptomatology.

Our observation of decreased ΔHbR in the contralateral dlPFC during and after stimulation aligns with previous evidence of rTMS‐induced distant effects, as demonstrated by interleaved high‐frequency rTMS/fMRI in healthy individuals^33^ and rTMS/fNIRS studies in treatment‐resistant depression.^66^, ^67^ All participant groups in our study exhibited comparable hemodynamic responses in the contralateral dlPFC, suggesting that iTBS may not elicit differential effects in distant regions, or that certain neurovascular coupling‐related properties (e.g., CBF) in depression are less affected in the right prefrontal area compared to the left. We found a significant reduction in global connectivity during iTBS. This indicates that network‐mediated effects of single‐session rTMS on remote brain regions are pronounced only during stimulation, whereas they diminish or disappear after stimulation.^25^, ^55^ The reduction in global connectivity likely reflects the immediate disruptive impacts of iTBS on cortices, which cause a temporary desynchronization of hemodynamic responses in prefrontal areas that resolve poststimulation.

This study has several limitations. First, TMS produces a brief, intense clicking noise that can stimulate the auditory system through air and bone transmission and interfere with the activity of the brain networks.^68^ Furthermore, the examination environment itself may trigger acute psychological stress in individuals with depression, leading to physiological and biochemical responses^69^ that may influence the present results. While a randomized sham‐controlled trial in healthy participants showed a significant increase in HbR change in the active group compared to the sham group (see Supplement Appendix S2), future studies in patient cohorts should incorporate sham coils for more robust control. Second, the sample size is moderate for such an inter‐individually highly heterogeneous sample. Third, our patient participants were primarily recruited from the community, with diagnoses initially made by different clinicians across various clinical settings. To enhance the reliability of the diagnoses and clinical information, all patient participants' diagnoses and clinical characteristics were verified by our research team. Fourth, we initially aimed to recruit age‐ and gender‐matched high‐risk healthy participants. However, the limited number of eligible individuals, coupled with challenges in verifying family histories of MDD, led to their exclusion for the time being. Lastly, vascular pathology in MDD may affect baseline vascular tone and, consequently, the stimulation‐induced hemodynamic response; future research with direct vascular measures is needed to better elucidate its role in modulating the hemodynamic response to iTBS in MDD.

Collectively, our results support existing clinical and preclinical studies that associate impaired neurovascular function in the prefrontal cortex with depression. Poststimulation ΔHbO in the targeted dlPFC appears to reflect genuine neuroplastic changes induced by iTBS. These responses not only correlate with the present depressive state but also indicate susceptibility to the risk of recurrence. Longitudinal studies are needed to evaluate the predictive value of iTBS/fNIRS in depression recurrence.

## Author contributions

M.J. and A.W.X. established the concurrent TMS/fNIRS setup, implemented the fNIRS data preprocessing pipeline, verified and analyzed the data. M.J. also drafted the manuscript. W.M.C. and P.P.Q. were responsible for participant recruitment. M.J., N.M.S., A.H.T., R.L.K., and S.X.W. performed the fNIRS scans. B.B.Z. advised on fNIRS signal processing, statistical analysis, and optode localization. T.T.L. designed the 3D‐printed TMS reference holder. D.W.C. conducted psychiatric assessments. F.P. contributed to editing and critical review of the manuscript. G.S.K. designed and supervised the study. All authors participated in the preparation of the manuscript and approved the final version.

## Funding statement

This work was supported by the General Research Fund (numbers 15,106,222 and 15,100,120) under the University Grants Committee of the HKSAR, as well as The Mental Health Research Center (numbers 0048822 and 0040786), The Hong Kong Polytechnic University.

## Disclosure statement

F.P. is a member of the International Scientific Advisory Board of Sooma (Helsinki, Finland); he has received speaker honoraria from Mag&More GmbH, the neuroCare Group (Munich, Germany), and BrainsWay Inc.; his lab has received support with equipment from neuroConn GmbH (Ilmenau, Germany), Mag&More GmbH, and BrainsWay Inc. All other authors report no financial interests or potential conflicts of interest.

## Supporting information


**Data S1.** STROBE Statement—checklist of items that should be included in reports of observational studies.

**Table S1.** Inclusion and exclusion criteria.
**Table S2.** Baseline hemodynamic measures in the bilateral dlPFC across groups.
**Table S3.** Correlations of hemoglobin responses with medication load index and symptom severity in patient groups.
**Table S4.** Characteristics of participants.
**Table S5.** Two‐Way ANOVA Comparison of bilateral dlPFC hemoglobin concentration changes: Active *vs*. Sham Groups.
**Figure S2.** The time course of hemodynamic responses in the stimulated and contralateral dorsolateral prefrontal cortex (dlPFC) across groups.

## Data Availability

The raw data and MATLAB code used in this study are available upon request from the corresponding author.
