# Supplementary material for: Acute prefrontal hemodynamic responses to intermittent theta burst stimulation correlate with current depression and episode recurrence: A cross‐sectional study
Source: Psychiatry Clin Neurosci. 2026 Apr 14;80(7):616–25. doi: 10.1111/pcn.70066 (PMC13332562; doi:10.1111/pcn.70066)
Supplement: Supplementary file 1 — Data S1. STROBE Statement—checklist of items that should be included in reports of observational studies. [file PCN-80-616-s001.docx]

STROBE Statement—checklist of items that should be included in reports of observational studies

|  | Item No. | Recommendation | Page  No. | Relevant text from manuscript |
| --- | --- | --- | --- | --- |
| **Title and abstract** | 1 | (*a*) Indicate the study’s design with a commonly used term in the title or the abstract | 2 | *Cross-sectional changes of hemodynamic responses and functional connectivity induced by a single session of intermittent theta burst stimulation over the left dorsolateral prefrontal cortex (dlPFC) were measured…* |
|  |  | (*b*) Provide in the abstract an informative and balanced summary of what was done and what was found | 2 | See Abstract section |
| Introduction | | | |  |
| Background/rationale | 2 | Explain the scientific background and rationale for the investigation being reported | 5 | See Introduction |
| Objectives | 3 | State specific objectives, including any prespecified hypotheses | 6 | *We applied…to investigate….; We hypothesized that…* |
| Methods | | | |  |
| Study design | 4 | Present key elements of study design early in the paper | 6 |  |
| Setting | 5 | Describe the setting, locations, and relevant dates, including periods of recruitment, exposure, follow-up, and data collection | 6 | *Adults with current or prior MDD were recruited through the Hong Kong Integrated Community Centre for Mental Wellness…;Data collection took place from October 8, 2023, to December 18, 2024.* |
| Participants | 6 | (*a*) *Cohort study*—Give the eligibility criteria, and the sources and methods of selection of participants. Describe methods of follow-up  *Case-control study*—Give the eligibility criteria, and the sources and methods of case ascertainment and control selection. Give the rationale for the choice of cases and controls  *Cross-sectional study*—Give the eligibility criteria, and the sources and methods of selection of participants | 6 | *Adults with current or prior MDD were recruited through the Hong Kong Integrated Community Centre for Mental Wellness…; see Table S1 in the Supplement for details on inclusion and exclusion criteria* |
|  |  | (*b*) *Cohort study*—For matched studies, give matching criteria and number of exposed and unexposed  *Case-control study*—For matched studies, give matching criteria and the number of controls per case | 6, 10 | *Age-and gender-matched healthy controls were recruited through advertisements; therefore, 39 MDD, 41 rMDD, and 41 HCs were included in the final analyses* |
| Variables | 7 | Clearly define all outcomes, exposures, predictors, potential confounders, and effect modifiers. Give diagnostic criteria, if applicable | 7, 8, 9 | See Method procedures |
| Data sources/ measurement | 8* | For each variable of interest, give sources of data and details of methods of assessment (measurement). Describe comparability of assessment methods if there is more than one group | 8, 9, 10 | See Method procedures, preprocessing of fNIRS signals, characterizing functional networks and statistical analysis sections |
| Bias | 9 | Describe any efforts to address potential sources of bias | 11 | *To reduce recall bias in retrospective reports, we categorized the number of previous episodes following Treadway (11) into three groups: single episode, 2~4 episodes, and five or more episodes* |
| Study size | 10 | Explain how the study size was arrived at | 9 | *The required sample size for this study was calculated using one-way analysis of variance (G*Power software, version 3.1.9.7.). Assuming a medium-to-large effect size (Cohen’s f = 0.3), a significance level (α) of 0.05, and a statistical power of 0.8, the minimum total sample size required was determined to be 111 participants (37 per group). Considering an anticipated ~ 20% dropout rate due to intolerable pain during stimulation observed in our previous work (26), we planned to enroll 47 participants in each group.* |

Continued on next page

| Quantitative variables | 11 | Explain how quantitative variables were handled in the analyses. If applicable, describe which groupings were chosen and why | 7, 8, 9, 10 | See Method |
| --- | --- | --- | --- | --- |
| Statistical methods | 12 | (*a*) Describe all statistical methods, including those used to control for confounding | 9, 10 | See Method |
|  |  | (*b*) Describe any methods used to examine subgroups and interactions | 10 | *In the case of hemodynamic changes (ΔHbO & ΔHbR) and global connectivity (wD̄ & D), linear mixed models were performed under the unstructured variance covariance structure…* |
|  |  | (*c*) Explain how missing data were addressed | 10 | *Nineteen individuals withdrew…therefore, 39 MDD, 41 rMDD, and 41 HCs were included in the final analyses* |
|  |  | (*d*) *Cohort study*—If applicable, explain how loss to follow-up was addressed  *Case-control study*—If applicable, explain how matching of cases and controls was addressed  *Cross-sectional study*—If applicable, describe analytical methods taking account of sampling strategy | 6 | *Age-and gender-matched healthy controls were recruited through advertisements;* |
|  |  | (*e*) Describe any sensitivity analyses | NA |  |
| Results | | | | |
| Participants | 13* | (a) Report numbers of individuals at each stage of study—eg numbers potentially eligible, examined for eligibility, confirmed eligible, included in the study, completing follow-up, and analysed | 10 | *A total of 1014 individuals registered for the study, and 143 participants were enrolled. Nineteen individuals withdrew (currently MDD = 8, remitted MDD, rMDD = 7, HCs = 4) due to intolerable pain perception during TMS test pulses (n = 12), newly identified TMS contraindications (n = 2), allergy to the fNIRS cap (n = 1), or personal reasons (n =3). One HC data set was lost because of a computer system failure. Data from three participants were excluded from further analysis due to bad signal quality, as described in the Methods; therefore, 39 MDD, 41 rMDD, and 41 HCs were included in the final analyses.* |
|  |  | (b) Give reasons for non-participation at each stage | 10 | Same as above |
|  |  | (c) Consider use of a flow diagram | / |  |
| Descriptive data | 14* | (a) Give characteristics of study participants (eg demographic, clinical, social) and information on exposures and potential confounders | Table 1 & 2 | *The participants' characteristics and distribution of psychotropic medication were summarized in Table 1 & 2.* |
|  |  | (b) Indicate number of participants with missing data for each variable of interest | Table 1 & 2 |  |
|  |  | (c) *Cohort study*—Summarise follow-up time (eg, average and total amount) | NA |  |
| Outcome data | 15* | *Cohort study*—Report numbers of outcome events or summary measures over time | NA |  |
|  |  | *Case-control study—*Report numbers in each exposure category, or summary measures of exposure | 10 | *39 MDD, 41 rMDD, and 41 HCs were included in the final analyses.* |
|  |  | *Cross-sectional study—*Report numbers of outcome events or summary measures | 10 | *39 MDD, 41 rMDD, and 41 HCs were included in the final analyses* |
| Main results | 16 | (*a*) Give unadjusted estimates and, if applicable, confounder-adjusted estimates and their precision (eg, 95% confidence interval). Make clear which confounders were adjusted for and why they were included | 10, 11 | *Post hoc tests showed a greater post-iTBS ΔHbO in the MDD group versus HCs (pcorrected < 0.001, 95% CI: 0.127 ~ 0.521)…This interaction was driven by the increased ΔHbR during stimulation in patient groups compared with HCs (MDD: pcorrected < 0·001, 95% CI = 0·115 ~ 0·600; rMDD: pcorrected = 0·036, 95% CI = 0·012~0·490)…* |
|  |  | (*b*) Report category boundaries when continuous variables were categorized | NA |  |
|  |  | (*c*) If relevant, consider translating estimates of relative risk into absolute risk for a meaningful time period | NA |  |

Continued on next page

| Other analyses | 17 | Report other analyses done—eg analyses of subgroups and interactions, and sensitivity analyses | 11 | *the relationship between dlPFC hemodynamic responses and the number of prior MDEs was analyzed by hierarchical multiple regression* |
| --- | --- | --- | --- | --- |
| Discussion | | | | |
| Key results | 18 | Summarise key results with reference to study objectives | 12 | See Discussion first paragraph |
| Limitations | 19 | Discuss limitations of the study, taking into account sources of potential bias or imprecision. Discuss both direction and magnitude of any potential bias | 14 | *This study has several limitations. First…..* *leading to physiological and biochemical responses (63) that may influence the present results…* |
| Interpretation | 20 | Give a cautious overall interpretation of results considering objectives, limitations, multiplicity of analyses, results from similar studies, and other relevant evidence | 12, 13, 14 | See Discussion |
| Generalisability | 21 | Discuss the generalisability (external validity) of the study results | 14 | *Patient participants were primarily recruited from the community* |
| Other information | |  | | |
| Funding | 22 | Give the source of funding and the role of the funders for the present study and, if applicable, for the original study on which the present article is based | 2 | *This work was supported by the General Research Fund (numbers 15106222 and 15100120) under the University Grands Committee of the HKSAR, as well as the Mental Health Research Center (numbers 0048822 and 0040786), The Hong Kong Polytechnic University.* |

*Give information separately for cases and controls in case-control studies and, if applicable, for exposed and unexposed groups in cohort and cross-sectional studies.

**Note:** An Explanation and Elaboration article discusses each checklist item and gives methodological background and published examples of transparent reporting. The STROBE checklist is best used in conjunction with this article (freely available on the Web sites of PLoS Medicine at http://www.plosmedicine.org/, Annals of Internal Medicine at http://www.annals.org/, and Epidemiology at http://www.epidem.com/). Information on the STROBE Initiative is available at www.strobe-statement.org.
