# Supplementary material for: Acute prefrontal hemodynamic responses to intermittent theta burst stimulation correlate with current depression and episode recurrence: A cross‐sectional study
Source: Psychiatry Clin Neurosci. 2026 Apr 14;80(7):616–25. doi: 10.1111/pcn.70066 (PMC13332562; doi:10.1111/pcn.70066)
Supplement: Supplementary file 2 — Table S1. Inclusion and exclusion criteria. Table S2. Baseline hemodynamic measures in the bilateral dlPFC across groups. Table S3. Correlations of hemoglobin responses with medication load index and symptom severity in patient groups. Table S4. Characteristics of participants. Table S5. Two‐Way ANOVA Comparison of bilateral dlPFC hemoglobin concentration changes: Active vs. Sham Groups. Figure S2. The time course of hemodynamic responses in the stimulated and contralateral dorsolateral prefrontal cortex (dlPFC) across groups. [file PCN-80-616-s002.docx]

**Supplementary material**

**Figure S1. Recruitment flow chart**

**
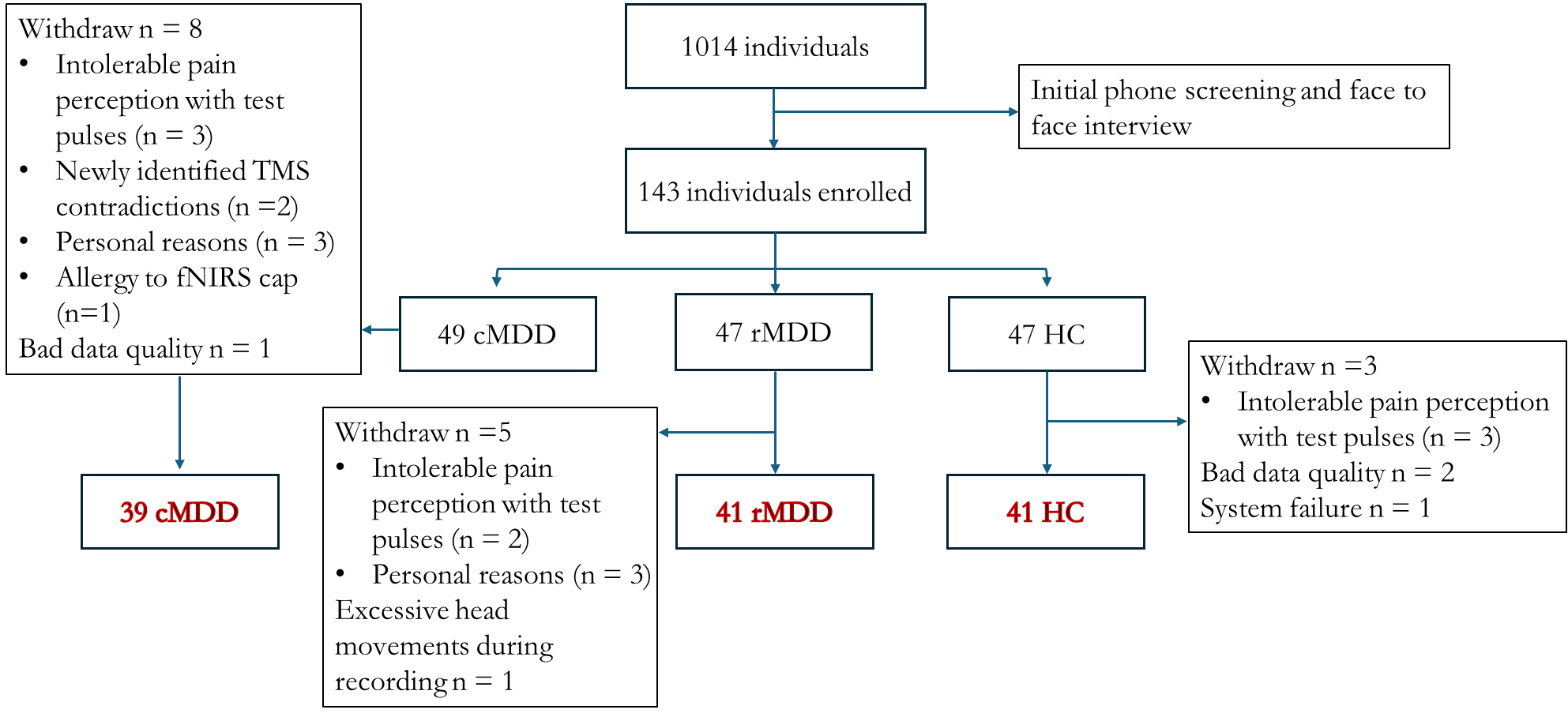
**

**Table S1. Inclusion and exclusion criteria**

| **Inclusion Criteria** | |
| --- | --- |
| Items | For HCs |
| 1 | Aged 18 to 65 |
| 2 | Healthiness based on history and psychiatric assessment |
| 3 | Patient Health Questionnaire-9 (PHQ-9) ≤ 4 |
| Items | For current MDD |
| 1 | Aged 18 to 65 |
| 2 | A clinical diagnosis of current unipolar depressive disorder by an experienced psychiatrist according to DSM-5 |
| 3 | No or stable (≥ 4 weeks) psychopharmacological medication |
| Items | For remitted MDD |
| 1 | Aged 18 to 65 (b); (c) no or stable (≥ 4 weeks) psychopharmacological medication. |
| 2 | A clinical diagnosis of recurrent depressive disorder by an experienced psychiatrist but currently in full remission (ICD 11, 6A71.7) according to results of the Mini International Neuropsychiatric Interview (MINI) and the Hamilton Depression Rating Scale-17, with a score ≤ 7 |
| **Exclusion criteria** | |
| Items | For HCs |
| 1 | TMS contraindications, such as epilepsy |
| 2 | Current or prior diagnoses of any psychiatric disorders |
| 3 | PHQ-9 > 4 |
| 4 | Not meeting full screening criteria for MDD |
| 5 | Substance or alcohol abuse |
| 6 | Thyroid function problems |
| 7 | Current or prior diagnoses of cancer |
| 8 | Other general medical conditions, such as diabetes, hypertension |
| 9 | Out of age range (18 – 65 years) |
| 10 | Family history of any psychiatric disorders |
| 11 | Age or gender unmatched |
| Items | For current MDD |
| 1 | TMS contraindications, such as epilepsy |
| 2 | Current or prior diagnoses including neurodevelopmental conditions, psychotic spectrum disorders, bipolar disorder, trauma-related disorders (including PTSD), dissociative disorders, or neurological conditions, such as stroke, traumatic brain injury, multiple sclerosis, or other brain disease |
| 3 | Not meeting full screening criteria for MDD |
| 4 | Substance or alcohol abuse |
| 5 | Current or prior electroconvulsive therapy or other brain stimulation intervention |
| 6 | Thyroid function problems |
| 7 | Current or prior diagnoses of cancer |
| 8 | Other general medical conditions, such as diabetes, hypertension |
| 9 | Out of age range (18 – 65 years) |
| Items | For remitted MDD |
| 1 | TMS contraindications, such as epilepsy |
| 2 | Current or prior diagnoses including neurodevelopmental conditions, psychotic spectrum disorders, bipolar disorder, trauma-related disorders (including PTSD), dissociative disorders, or neurological conditions, such as stroke, traumatic brain injury, multiple sclerosis, or other brain disease |
| 3 | Not meeting full screening criteria for remitted MDD |
| 4 | HAMD-17 > 7 |
| 5 | Substance or alcohol abuse |
| 6 | Current or prior electroconvulsive therapy or other brain stimulation intervention |
| 7 | Thyroid function problems |
| 8 | Current or prior diagnoses of cancer |
| 9 | Other general medical conditions, such as diabetes, hypertension |
| 10 | Out of age range (18 – 65 years) |

**Table S2. Baseline hemodynamic measures in the bilateral dlPFC across groups**

| Regions | Controls | cMDD | rMDD | F-statistics | P value |
| --- | --- | --- | --- | --- | --- |
| Left-dlPFC_HbO (micro-mol) | 0.019 (0.231) | -0.102 (0.222) | -0.046 (0.141) | 3.628 | 0.030 |
| Left-dlPFC_HbR (micro-mol) | -0.070 (0.142) | -0.134 (0.267) | -0.081 (0.223) | 0.718 | 0.490 |
| Right-dlPFC_HbO (micro-mol) | 0.030 (0.152) | 0.004 (0.134) | -0.003 (0.141) | 0.568 | 0.568 |
| Right-dlPFC_HbR (micro-mol) | 0.016 (0.123) | 0.011 (0.128) | 0.019 (0.116) | 0.043 | 0.958 |

Data expressed as mean (SD)

| **Table S3. Correlations of hemoglobin reponses with medication load index and symptom severity in patient groups** | | | | | |
| --- | --- | --- | --- | --- | --- |
| covariate | hemoglobin responses | MDD (n=38) | | rMDD (n=40) | |
|  |  | r | p value* | r | p value* |
| medication load index | left dlPFC HbR during iTBS | -0.205 | 0.218 | -0.131 | 0.421 |
|  | left dlPFC HbO post iTBS | 0.11 | 0.512 | -0.105 | 0.518 |
| symptom severity (measured by HAMD-17) | left dlPFC HbR during iTBS | 0.075 | 0.656 | 0.098 | 0.544 |
|  | left dlPFC HbO post iTBS | 0.132 | 0.43 | 0.099 | 0.538 |
| * Calculated by Spearman-Rho correlation coefficients | | | | | |

**Appendix 1. fNIRS data preprocessing**

To thoroughly characterize the hemodynamic response elicited by iTBS, both HbO and HbR were utilized as primary indicators. Each channel’s raw data in every dataset was visually reviewed, and channels with inadequate signal quality were excluded based on predefined dRange criteria (minimum 500 μV, maximum 1 V) specific to the NIRxBorealis system, as well as cardiac power assessed via spectral analysis. The remaining raw signals were converted into optical density and subsequently transformed into concentration changes of HbO (ΔHbO) and HbR (ΔHbR) according to the modified Beer-Lambert Law. Separate analyses were conducted for ΔHbO and ΔHbR. For each standard channel, the most correlated short-separation channel (SSC) was chosen as a regressor for short-separation regression throughout the entire measurement period, using a least-squares method (1-4) to suppress superficial tissue and motion artifacts.

To further reduce physiological and movement-related noise, independent component analysis (ICA) was applied to each participant’s data. Prior to ICA, principal component analysis (PCA) was used to compress the signal to the number of components that captured at least 99% of the variance (5, 6). ICA decomposition was performed with FastICA v2.5 ([www.cis.hut.fi/projects/ica/fastica/](http://www.cis.hut.fi/projects/ica/fastica/)) using these settings: number of independent components set to the number of PCs, maximum iterations = 10,000, epsilon = 1e-5, “deflation” approach, random initialization, “skew” nonlinearity, and both finetune and stabilization enabled (6). U-shaped or inverted-U-shaped independent components (ICs) were identified based on: (a) displaying a sustained (40–60 s) U or inverted-U-shaped fluctuation during the resting period; and (b) occurring in bilateral marginal channels covering the lateral inferior frontal gyrus (5). For transient fluctuations, ICs were selected if (a) their time series showed multiple peaks within short intervals, coinciding with each 2-second iTBS burst, and (b) they appeared in channels over the stimulated hemisphere.

Despite ICA, some motion artifacts might remain, potentially biasing the estimation of the true hemodynamic response and leading to statistical errors (7). To address this, we performed wavelet-based motion artifact correction (α = 0.1) (8) and applied a low-pass filter with a high-frequency cut-off at 0.09 Hz to further eliminate residual movement and physiological noise (including heartbeats, respiration, and Mayer waves), while preserving low-frequency signals that reflect the overall hemodynamic trend throughout the session. Finally, baseline correction was implemented by subtracting the mean value from the 3-minute pre-iTBS resting period. The mean hemoglobin concentration changes during (3 minutes) and after (3 minutes) iTBS were then calculated for each phase, and these values were included in the subsequent statistical analyses.

**Appendix 2. Graph theory metrics calculated to describe the topology of functional networks**

1. Normalized connection strength for each node: $wD_{i}= \frac{1}{N} \sum_{j=1}^{N} w_{ij}$
2. Normalized global connection strength: $w\bar{D}= \frac{1}{N-1} \sum_{i} wDi$
3. Normalized local node degree: $D_{i}= \frac{1}{N} \sum_{j=1}^{N} b_{ij}$
4. Normalized global node degree: $\bar{D}= \frac{1}{N-1} \sum_{i} Di$

*N* is the set of all nodes in the network, used for normalization. *wᵢⱼ* is the weighted adjacency matrix that quantifies the connection strength between nodes *i* and *j*. *b_ij_* is the connection status between *i* and *j*: *b_ij_ =* 1 when link (i, j) is present and *b_ij_ =* 0 otherwise.

**Appendix 3. A randomized double-blind sham-controlled trial**

32 TMS-naïve healthy controls (mean age: 26.53 ± 5.668 years; mean education: 17.5 ± 1.65 years; 10 males, 22 females) were included. All participants were free of neurological or psychiatric disorders, had no family history of psychiatric conditions, and scored ≤ 4 on the PHQ-9. They were randomly assigned to either the active iTBS group or the sham iTBS group (participant characteristics are detailed in Table S3). The sham iTBS condition did not deliver actual stimulation but simulated auditory noise to ensure a comparable experimental environment. The experimental setup was identical to that in the manuscript, except a MagVenture Cool-B65 A/P sham coil was used. Data preprocessing followed the established pipeline

Two-way ANOVA revealed a significant Group * Time interaction effect for HbR changes in the stimulated dlPFC (F = 6.041, p = 0.004, η² = 0.172), along with significant Time (F = 10.066, p < 0.001, η² = 0.258) and Group effects (F = 11.342, p = 0.002, η² = 0.281). Bonferroni post hoc analysis showed HbR changes were significantly greater in the active group compared to sham during stimulation (p*_corrected_* = 0.003) and post-stimulation (p*_corrected_* = 0.027) (see Table S4). The time course of hemodynamic responses in the stimulated and contralateral dlPFC across groups is shown in Figure S2.

**Table S4. Characteristics of participants**

| Characteristics | Active Group | Sham Group | P value |
| --- | --- | --- | --- |
| Age (mean ± SD) | 26.44 ± 4.52 | 26.63 ± 6.78 | 0.927 |
| Gender (No. of females, %) | 11 (68.75) | 11 (68.75) | 1 |
| Education Year | 17.69 ±1.92 | 17.31 ± 1.35 | 0.528 |
| Marrital Status (No. of married, %) | 1 (6.25) | 3 (18.75) | 0.6 |
| Employment Status (No. of unemployment, %) | 0 (0) | 1 (6.25) | 1 |
| Race (No. of Chinese, %) | 16 (100) | 16 (100) | 1 |
| PHQ-9 (mean ± SD) | 1.56 ± 1.50 | 1.88 ± 1.59 | 0.572 |

**Table S5. Two-Way ANOVA Comparison of bilateral dlPFC hemoglobin concentration changes: Active vs. Sham Groups**

| Brain area_Hb | Group Effect | | Time Effect | | Group * Time Interaction Effect | | Post hoc analysis |
| --- | --- | --- | --- | --- | --- | --- | --- |
|  | F | p | F | p | F | p |  |
| IDLPFC_HbO | 0.419 | 0.522 | 0.101 | 0.904 | 2.123 | 0.129 | / |
| lDLPFC_HbR | 11.342 | 0.002 | 10.066 | < 0.001 | 6.041 | 0.004 | during stimulation: active > sham (pcorrected = 0.003) post stimulation: active > sham (pcorrected = 0.027) |
| rDLPFC_HbO | 0.557 | 0.461 | 0.129 | 0.879 | 0.363 | 0.697 | / |
| rDLPFC_HbR | 1.009 | 0.323 | 1.076 | 0.348 | 0.676 | 0.512 | / |

**Figure S2. The time course of hemodynamic responses in the stimulated and contralateral dorsolateral prefrontal cortex (dlPFC) across groups**

**Reference**

1. Saager RB, Berger AJ. Direct characterization and removal of interfering absorption trends in two-layer turbid media. J Opt Soc Am A Opt Image Sci Vis. 2005;22(9):1874-82.

2. Scholkmann F, Metz AJ, Wolf M. Measuring tissue hemodynamics and oxygenation by continuous-wave functional near-infrared spectroscopy--how robust are the different calculation methods against movement artifacts? Physiol Meas. 2014;35(4):717-34.

3. Saager RB, Telleri NL, Berger AJ. Two-detector Corrected Near Infrared Spectroscopy (C-NIRS) detects hemodynamic activation responses more robustly than single-detector NIRS. Neuroimage. 2011;55(4):1679-85.

4. Fabbri F, Sassaroli A, Henry ME, Fantini S. Optical measurements of absorption changes in two-layered diffusive media. Phys Med Biol. 2004;49(7):1183-201.

5. Zhu H, Xu J, Li J, Peng H, Cai T, Li X, et al. Decreased functional connectivity and disrupted neural network in the prefrontal cortex of affective disorders: A resting-state fNIRS study. J Affect Disord. 2017;221:132-44.

6. Zhang H, Zhang YJ, Lu CM, Ma SY, Zang YF, Zhu CZ. Functional connectivity as revealed by independent component analysis of resting-state fNIRS measurements. Neuroimage. 2010;51(3):1150-61.

7. Di Lorenzo R, Pirazzoli L, Blasi A, Bulgarelli C, Hakuno Y, Minagawa Y, et al. Recommendations for motion correction of infant fNIRS data applicable to multiple data sets and acquisition systems. Neuroimage. 2019;200:511-27.

8. Brigadoi S, Ceccherini L, Cutini S, Scarpa F, Scatturin P, Selb J, et al. Motion artifacts in functional near-infrared spectroscopy: a comparison of motion correction techniques applied to real cognitive data. Neuroimage. 2014;85 Pt 1(0 1):181-91.
